# Spectral Reflectance Reconstruction Using Fuzzy Logic System Training: Color Science Application

**DOI:** 10.3390/s20174726

**Published:** 2020-08-21

**Authors:** Morteza Maali Amiri, Sergio Garcia-Nieto, Samuel Morillas, Mark D. Fairchild

**Affiliations:** 1 Munsell Color Science Laboratory, Rochester Institute of Technology, New York, NY 14623, USA; mm2391@rit.edu (M.M.A.); mdf@mail.rit.edu (M.D.F.); 2 Instituto de Automática e Informática Industrial, Universitat Politècnica de València, 46022 Valencia, Spain; sgnieto@isa.upv.es; 3 Instituto Universitario de Matemática Pura y Aplicada, Universitat Politècnica de València, 46022 Valencia, Spain

**Keywords:** spectral recovery, CIEXYZ, RGB, fuzzy logic, fuzzy logic inference systems

## Abstract

In this work, we address the problem of spectral reflectance recovery from both CIEXYZ and RGB values by means of a machine learning approach within the fuzzy logic framework, which constitutes the first application of fuzzy logic in these tasks. We train a fuzzy logic inference system using the Macbeth ColorChecker DC and we test its performance with a 130 sample target set made out of Artist’s paints. As a result, we obtain a fuzzy logic inference system (FIS) that performs quite accurately. We have studied different parameter settings within the training to achieve a meaningful overfitting-free system. We compare the system performance against previous successful methods and we observe that both spectrally and colorimetrically our approach substantially outperforms these classical methods. In addition, from the FIS trained we extract the fuzzy rules that the system has learned, which provide insightful information about how the RGB/XYZ inputs are related to the outputs. That is to say that, once the system is trained, we extract the codified knowledge used to relate inputs and outputs. Thus, we are able to assign a physical and/or conceptual meaning to its performance that allows not only to understand the procedure applied by the system but also to acquire insight that in turn might lead to further improvements. In particular, we find that both trained systems use four reference spectral curves, with some similarities, that are combined in a non-linear way to predict spectral curves for other inputs. Notice that the possibility of being able to understand the method applied in the trained system is an interesting difference with respect to other ’black box’ machine learning approaches such as the currently fashionable convolutional neural networks in which the downside is the impossibility to understand their ways of procedure. Another contribution of this work is to serve as an example of how, through the construction of a FIS, some knowledge relating inputs and outputs in ground truth datasets can be extracted so that an analogous strategy could be followed for other problems in color and spectral science.

## 1. Introduction

Having access to spectral information is of great importance and usefulness; being considered object finger prints is only one of the indications attesting to its applicability. Enabling to reproduce the color of the object under different illumination types is yet another indication of the importance of the spectral data. In fact, the spectral reflectance is utilized in a wide variety of applications. For example, color matching used in the textile industry often utilizes the Allen algorithm; this algorithm needs spectral reflectance of the object as input [1]. Also, the prediction of object appearance variation under a wide range of illuminants is possible through having access to the spectral reflectance of the object. This approach has been applied in computer aided design (CAD) [1], illumination design for museums [2] or characterization of the degradation process of the varnishes on artworks [3]. In addition, applications as diverse as realistic image synthesis using computer graphics [4] or the estimation of surface temperature exposed to sunlight [5], also rely on the input of spectral information.

Nonetheless, the spectral data cannot be easily accessed. For instance, there should at least be a spectrophotometer or a hyperspectral camera for the spectral data to be accessed, both of which could be prohibitively high-priced or inaccessible due to other reasons such as impossibility to embed in portable devices, for instance. Their corresponding colorimetric data, though, such as nonstandard RGB and standard CIEXYZ data, can be accessed at a much lower price. Despite attaining the colorimetric data from their corresponding spectral information is straightforward, the reverse process, computation of the spectral reflectance from their colorimetric data is an ill-posed problem.

Consequently, spectral reflectance estimation from colorimetric data has been the aim of a number of different studies [6,7,8,9,10,11,12,13,14]. The majority of the methods until now have drawn upon linear approaches such as Principal Components Analysis (PCA) which traces back to the reasoning that spectral reflectance of non-fluorescent objects is typically a smooth function of wavelength [1,15]. Many different modifications of PCA have also been observed; the use of other methods such as interpolation, non-negative matrix factorization, Pseudo-Inverse and so on is also customary [16].

All the above-mentioned methods have used CIEXYZ tristimulus values as their input. There exists a wide range of procedures to estimate the spectra in this way, such as PCA: non-negative matrix factorization, and others which upside concerns that factors such as noise and uncertainty in the system does not influence the final recovery accuracy. On the other hand, the use of RGB input values for the spectral recovery process is also of interest, despite the fact that it is not a standard colorimetric space [17,18,19]. Many of the color reproduction and acquisition devices would use RGB instead of CIEXYZ. Therefore, a more practical spectral recovery should input RGB data to obtain the recovery. In this context, it has been shown that using separate paths, one for spectral and the other for colorimetric color reproduction, is the most efficient way when it comes to using digital cameras in spectral recovery attempts [17]. It has also been reported that the use of colored filters in front of digital cameras enhances the spectral recovery accuracy significantly [18]. Also, Cao et al., recently, came up with their own formula for spectral recovery in which only learning samples close to the testing samples in terms of color difference are used [19]. Amiri and Fairchild approached this problem from a different angle [20]: they stated that cameras and humans possess some kind of variability in their spectral sensitivity functions. They demonstrated how this variability can be taken advantage of in the spectral recovery process by combining different types of camera RGB responses with each other.

In this work we address the problem of spectral recovery using fuzzy logic tools. Fuzzy logic is a well-known theory widely used in many areas of science and engineering for its capability to represent knowledge and deal with imprecise data and uncertainty in both knowledge and data [21]. Also, it is proven that fuzzy logic inference systems (FIS) are universal function approximators [22]. Usually, FIS are built from expert knowledge expressed in terms of imprecise implication rules. Thus, FIS allow to make a computational algorithm from uncertain information [23]. However, recently, several approaches have studied how to learn the implication rules that relate inputs and outputs from data, in a kind of reverse engineering process [24]. In this work we approach the spectral recovery from this point of view. Using a database of spectral data and their corresponding XYZ and RGB values we use a training algorithm to learn the fuzzy rules that better relate XYZ/RGB data with spectral data. Then, the algorithm is tested against an independent database, which is also used to compare performance against the classical spectral recovery approaches in each case. Finally, we describe the fuzzy rules that the system uses, which provides insightful information and physical meaning about how the different spectral data are related to XYZ/RGB data. Despite FIS are not the only universal approximators and there exists other machine learning frameworks like support vector machines, Gaussian mixture models, or the more recent convolutional neural networks used for deep learning, all these operate as ’black boxes’ and FIS are the only option that allows to extract the learned knowledge in a comprehensive way. This is an important upside for FIS with respect to other options as it allows to assign a physical and/or conceptual meaning to its performance that allows not only to understand the procedure applied by the system but also it provides insight that in turn might lead to further improvements.

In the next section of the paper we briefly describe the basics of fuzzy logic and the method used to train a fuzzy logic inference system from groundtruth data. In Section 3 we show the experimental results separately for training with XYZ and RGB data along with a description of the obtained systems and performance comparison in terms of RMSE and CIEDE2000 against reference methods: the PCA method for XYZ data and the Pseudo-Inverse and Cao’s method for RGB data, as well as general discussion about the obtained methods. Last, in Section 4 we draw some conclusions.

## 2. Fuzzy Logic Inference Modeling Tool

Fuzzy logic arises from the classical logic shortcomings when dealing with uncertainty and imprecise reasoning. In fact, classical logic reasoning is based on the use of *IF-THEN* implication rules composed by an antecedent and a consequent [21]. When the antecedent is known true, the deduction process allows to infer that the consequent is also true. For instance, a classical logic rule can be: *IF (A AND B) OR C THEN D*. With this rule, *D* would be deduced to be true only when facts *A* and *B* are true or when fact *C* is true.

The main limitation of classical logic concerns those cases when certainty of facts cannot be expressed in a crisp way and when knowledge is expressed in imprecise terms. The latter are closely related to the form in which human ways of reasoning are expressed. For instance, a fuzzy implication rule could have the form: *IF X is high AND Y is low AND Z is low THEN W1 is high*. The fuzzy logic paradigm provides the needed tools to deal with this kind of implication rule. Here, not only the relation between the output W1 and the inputs X, Y, Z is imprecise, but also the facts in the rules are linguistic variables, namely, *X is high*, *Y is low*, *Z is low*, and *W1 is high*, which are impossible to be judged true or false from a crisp point of view. Instead, they are associated to degrees of certainty. These degrees of certainty are values in [0,1] computed with fuzzy membership functions from the numerical values of the inputs X,Y,Z in a process called *fuzzification* [21]. The certainty of the whole antecedent of the rule is then computed from the certainty of the linguistic variables by using a series of operators including t-norms and s-norms to deal with conjunction (AND) and disjunction (OR) operations, among others. Next, using a procedure called *fuzzy inference*, the certainty of the consequent is deduced from the one of the antecedents (*W1 is high* in our example). Last, comes the process of obtaining a numerical value for the output W1 from the degrees of certainty of the linguistic variable *W1 is high*, and others where W1 could be involved and that could be consequent of other rules such as: *W1 is low* or *W1 is medium*. This process is called *defuzzification* and is not unique, that is, there are several numerical methods to carry it out [22]. The set of rules along with the choice made concerning membership functions and the rest of the operators compose a fuzzy inference system (FIS).

A very important point in favor of FIS is that they have been proven to be universal function approximators. That is, any relation between inputs and outputs can be modeled using FIS to any degree of precision provided that the appropriate implication rules, membership functions, inference operators and defuzzification mechanism are properly selected [22]. So, they can be used to solve almost any function approximation problem and in particular to solve the spectral curve recovery problem addressed in this work.

**Figure 1:**
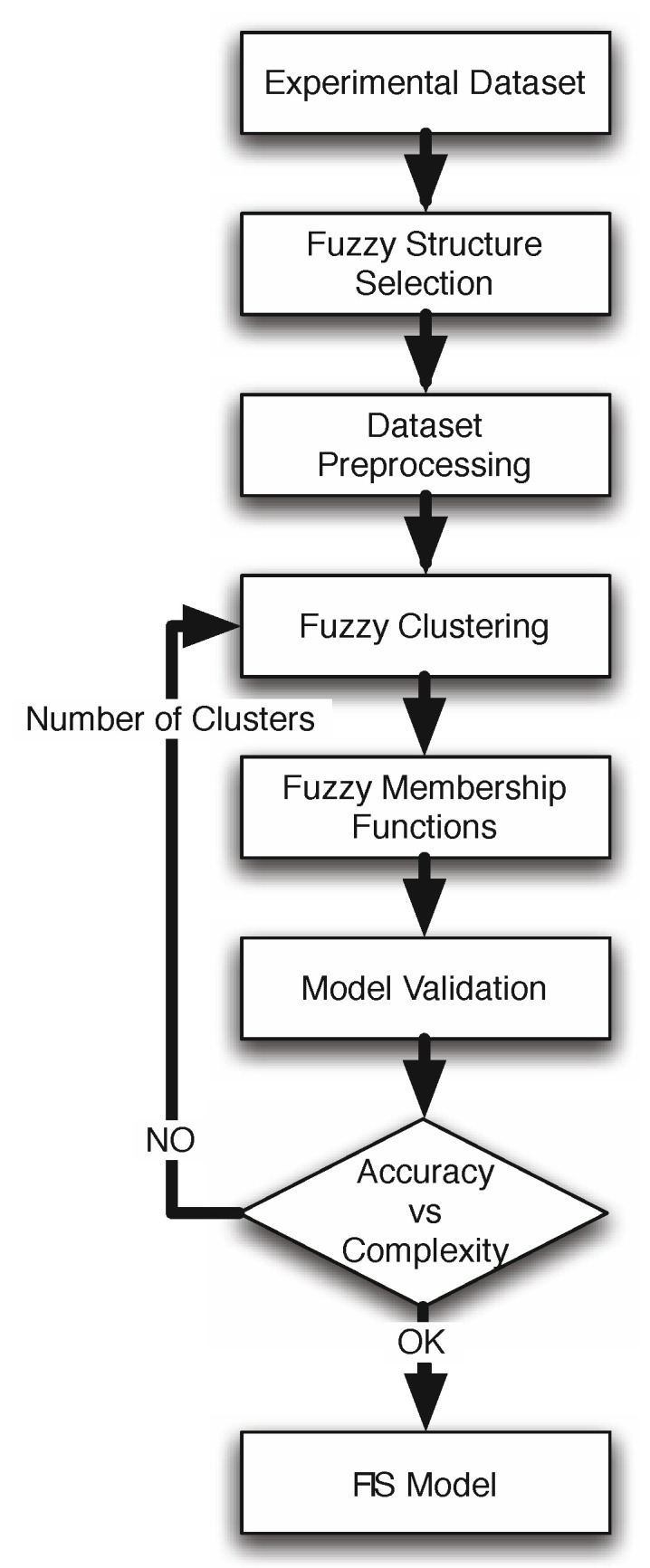
Modeling procedure.

Usually, FIS are built based on expert knowledge. That is, on the existence of a human solution for the problem to be solved. However, in many cases this expert knowledge is not available but, instead, there exists a set of ground truth data that relates the inputs and outputs of the desired system. Therefore, recently, it has been studied how a FIS can be built using ground truth data. That is, how using these data a set of implication rules and fuzzy membership functions can be learned to relate input and outputs. In turn, the knowledge represented in the fuzzy rules learned by the system can be used to better understand the problem and the solution learned. In this work we use this approach to address the problem of spectral curve recovery from XYZ and RGB data. In particular, we use the fuzzy logic modeling approach proposed by R. Babuska in [25,26] and made public through [27], where the main idea is to apply fuzzy clustering over the space of variables [28,29,30]. The goal is to identify subspaces with similar features where (non)linear submodels will be characterized. Those submodels are part of a global non-linear model that combines all the (non)linear models using fuzzy rules. The identification method obtains a matrix membership that expresses the degree of fulfillment of each fuzzy rule. Later, the membership function for each antecedent variable is directly obtained from the projection of that membership matrix. Figure 1 schematically shows the main steps in the identification procedure [26], which is iterative in its nature. In a typical modeling session, some of the steps may be repeated for different choices of the various parameters.

As we mentioned before, usually, fuzzy logic is based on a behavioural knowledge of the process, while expert knowledge expressed verbally and then translated into a collection of *IF-THEN* rules [31]. Parameters in this structure (membership functions, consequents, etc.) can be fine-tuned using data, or with first principle equations. However, the used method is included in the fuzzy data-driven techniques [25,26,30,31,32], where no prior knowledge about the system under study is initially used to formulate the rules; and a fuzzy model is constructed using numerical data only. It is expected that the extracted rules and membership functions can provide an a posteriori interpretation of the system behavior.

The different steps from Figure 1 are outlined below:

- ***Experimental Dataset***: This is an important initial step for any identification method, since it determines the information content of the identification data set. In this work we use CIEXYZ/RGB as inputs and Spectral data as desired outputs.
- ***Fuzzy Structure Selection***: In this step, the relevant variables with respect to the aim of the modeling are determined—based on prior knowledge regarding the process, or by trial and error. Also, the structure is selected as a Takagi–Sugeno (TS) fuzzy model [33]. The TS structures define the fuzzification/defuzzification method as well as the use of a fuzzy proposition in the antecedents and crisp functions in the consequents [34].
- ***Dataset Preprocessing***: Usually, the normalization of datasets helps the clustering process [31]. However, in this particular case no normalization has been applied, using the original dataset directly.
- ***Fuzzy Clustering***: The main goal is to obtain a partition of the dataset in a set of clusters, using fuzzy clustering. The designer determines a priori the number of clusters to obtain, and the clusters defining the number of local linear submodels of the fuzzy model, since the antecedent and consequent terms are obtained using the clustering results. The partition is obtained by using the Gustalfson-Kessel (GK) algorithm, first introduced in [28], and used frequently in clustering tasks [25,26,31]. This algorithm computes the fuzzy partition of the dataset to obtain the antecedent membership functions and consequent parameters. It is very important to point out that in a multiple input and multiple output system, such as our case, each different output can be associated to a different number of rules and, consequently, to a different clusterization of the input data space. This is a point that we study in detail in the next section and that is of key importance to the problem of spectral recovery. In particular, it is interesting to find out whether all outputs could be predicted using the same number of rules or not and, so, whether the clusterization used can be common to all outputs, which could lead to a more meaningful model than otherwise.
- ***Fuzzy Membership Functions***: To obtain the membership functions, the multidimensional fuzzy sets defined point-wise by the GK algorithm can be projected onto the space variables, using the point-wise projection operation introduced in [35]. The point-wise defined fuzzy sets obtained after the projection are approximated by a suitable parametric function, in order to be able to obtain a continuous function over the range of the regression variables. This method to obtain the membership functions is defined as Product Space Clustering and was first introduced in [26].
- ***Model Validation***: Once the whole set of parameters for the fuzzy model are defined (antecedents, consequents, etc.), the next step is to validate the result using a different, independent, set of inputs (RGB/XYZ). The testing set is inputted to the trained system to obtain predictions of the associated output (spectral data). The performance index used to define the degree of accuracy obtained by the model is the root mean squared error (RMSE) [36]. When the validation step determines a poor value of index RMSE, or an unbalanced result for the different outputs, the designer should reject this model and return to the structure model selection step.
- ***Accuracy vs. Complexity***: Finally, the design procedure ends with a qualitative study for the trade-off between accuracy vs. complexity of the fuzzy model. In this paper, the number of fuzzy rules is the parameter that determines the level of accuracy and complexity. A high number of rules allows the system for representing more information and input/output relations where a lower number limits its performance. However, an excessive number of rules can lead the system to training data overfitting which should be avoided, meaning that the number of rules should be limited and ideally, kept as low as necessary.
  Obviously, this procedure is not automatic and exact, but it helps to select the most appropriate fuzzy model from the designer point of view. However, the authors consider as a future work tackling this step as a multiobjective optimization problem, where the goal is to obtain a Pareto frontier for the accuracy-complexity problem [37].

Finally, It is interesting to note that the modeling tool [27] allows to extract the membership functions and the fuzzy rules that the system has learned, which provide invaluable information to understand how the system associates inputs and outputs and insightful information to advance towards better solutions for the problem. We will also discuss this in the next section.

## 3. Fiss Training and Test

**Figure 2:**
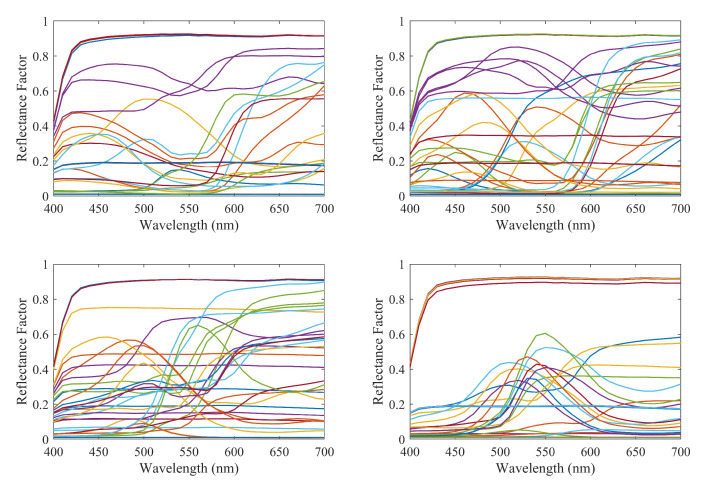
Spectral curves of the samples in the training dataset.

**Figure 3:**
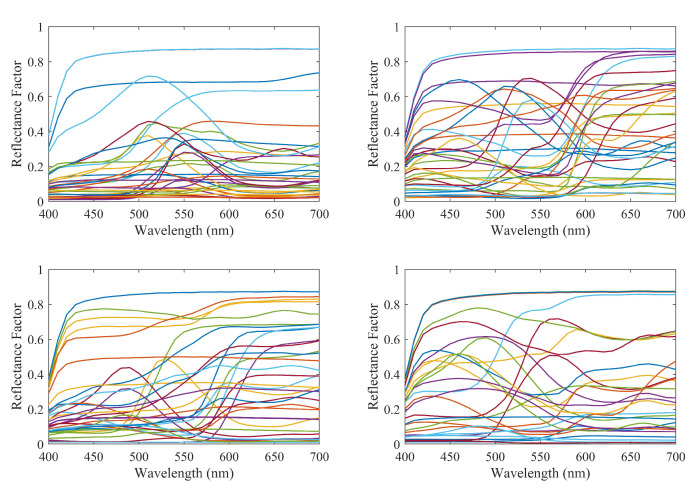
Spectral curves of the samples in the testing dataset.

As described above, we use two different independent datasets of spectral curves for training (Figure 2) and testing (Figure 3), respectively. In both sets the spectral information given by 31 coefficients associated to 31 wavelengths from 400 to 700 nm inclusive with a setup of 10 nm is associated to the corresponding XYZ and RGB data. The Macbeth ColorChecker dataset including 140 patches was used for training whereas a 130 sample target dataset made out of Artist’s paints was used as testing samples. In order to obtain the RGB values of the testing and learning samples, a Nikon D40 digital camera was used and two pictures were taken of the testing and learning samples in a light booth under a D65 light source using the raw camera mode to avoid problems with the setting of the choice of the color space. The mean patch values of the samples were then calculated and used as the camera RGB response. The camera photometric response was also linearized before using the RGB responses using the approach proposed in [38].

To assess the performance of the spectral data predicted by our FIS for different parameter settings as well as to compare with the classical methods in each case we use the RMSE and the CIEDE2000 color difference formula between the predicted data and the actual data in the testing dataset. In the case of the CIEXYZ, the results of the developed FIS spectral recovery performance is compared to the Principal Component Analysis (PCA) method [15], a successful classical procedure often used in this area. For RGB data, the method proposed by Cao’s et al. [19] and the Pseudo-Inverse method [20] are used as comparison references.

### 3.1. Spectral Recovery from Ciexyz Values

To carry out the FIS training from CIEXYZ data, we proceed in a series of steps in order to sequentially study the different parameter settings and configurations possible. This is a more practical approach than studying all possible configuration at a time, which would make the number of possibilities to study being high and practically inaccessible. There are many options within a FIS configuration that could be studied and compared: from t-norms and s-norms choices and shapes of membership functions to defuzzification, the number of options is vast. In our study, for practical reasons, we restrict the study to those parameters that, in our experience, are more likely to have a stronger impact on global system performance.

**Figure 4:**
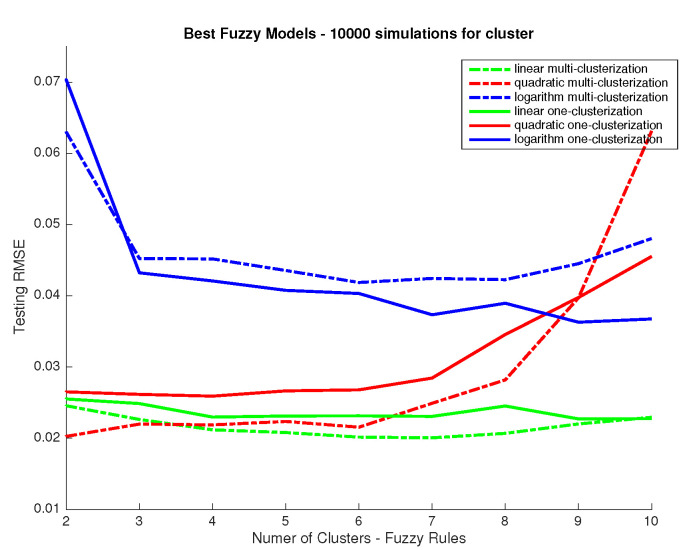
RMSE between predictions from the Fuzzy Logic Inference System (FIS) and desired outputs in the CIEXYZ training dataset (y axis) vs. number of clusters used (x axis). Legend indicates for each plot if the same input clusterization is used for all outputs or not.

**Figure 5:**
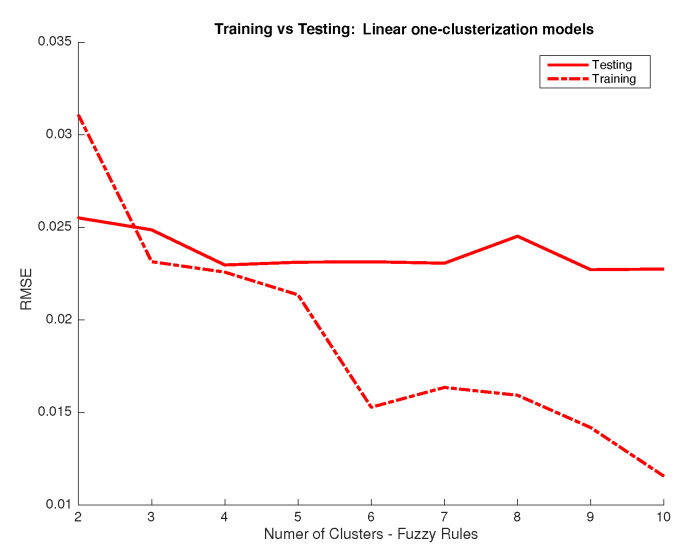
RMSE between predictions from the FIS system using the same clusterization for all inputs and linear activation functions and desired outputs in the CIEXYZ training and testing datasets (y axis) vs. number of clusters used (x axis).

The most important parameter we study is the number of rules to be used in the system, which equals the number of clusters to split the dataset: the system we aim to develop is a multiple input multiple output (MIMO) one. The problem could be seen as well as a series of multiple input and single output (MISO) systems. This latter case would allow a different input clusterization per output and so a different way to approach the response associated to each wavelength. So, first, we aim to determine if all outputs (31 wavelength responses) could be predicted using the same number of rules and then have only one MIMO system using the same input clusterization for all outputs or, in other case, we should use a series of MISO systems using different clusterization and rules for different outputs. To answer this we performed 10,000 training simulations considering a fixed number of clusters for all inputs (one MIMO system) and a variable number of clusters per output (several MISO systems) for different clustering levels (number of clusters used) and we compared the performance in terms of RMSE, which we show in Figure 4. In addition, we considered the use of linear response functions for the output and other alternatives such as logarithmic and quadratic responses. From the results in Figure 4 we can conclude that linear responses provide better performance. Also, the improvement obtained when considering different clusters (and so rules) for the different outputs is marginal. As it is a priority for us to develop a model that we can relate to a physical/conceptual meaning, we prefer using the same number of clusters and rules for all outputs as this would ease the explanation of how the system works and increase the meaningfulness of the system. Once that we have decided to use the same number of clusters for all outputs we study the performance depending on the number of clusters. As we can see in Figure 5, performance is not improved for the testing dataset when using more than four clusters, which means that any improvement above that number of clusters for the training dataset should be related to data overfitting. Therefore, we conclude that using four clusters is a good choice.

Once these parameters are set we train and build the FIS for spectral recovery from CIEXYZ data using four clusters per output and linear responses. Now, we analyze the FIS built to explain how it is working and to provide a physical meaning for the method obtained.

**Figure 6:**
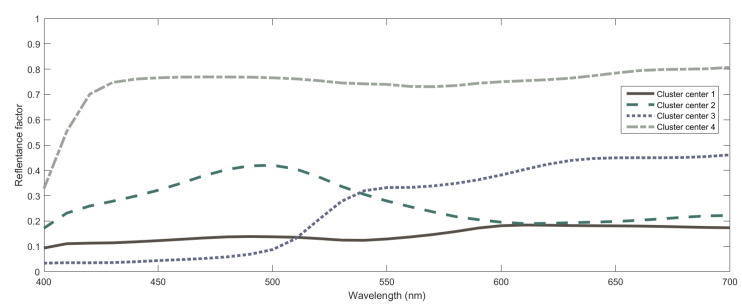
Spectral curves associated to cluster centers obtained in the FIS using CIEXYZ data. For each center, the curve is plotted using the RGB coordinates associated to the color represented by the curve itself for illustrative purposes.

The first step in this analysis is to have a look at the center of the four clusters determined (see Table 1). Understanding what the clusters represent is important as they will serve as the basis to carry out the spectral curve recovery and explain how the method acts in terms of the physical magnitudes of the inputs. We can see from this table that cluster 1 is associated to low XYZ values, cluster 2 to low-medium XYZ values, cluster 3 to medium XY values and very low Z values and cluster 4 to high XYZ values. It is interesting to note here that cluster centers are very important when predicting outputs related to inputs that are very close to them. In these cases, outputs are predicted using linear interpolation based on the cluster center outputs (as we selected linear output prediction above). Outputs (spectral curves) related to cluster centers are plotted in Figure 6. These are the curves that are combined to predict the spectral curves for other inputs.

**Figure 7:**
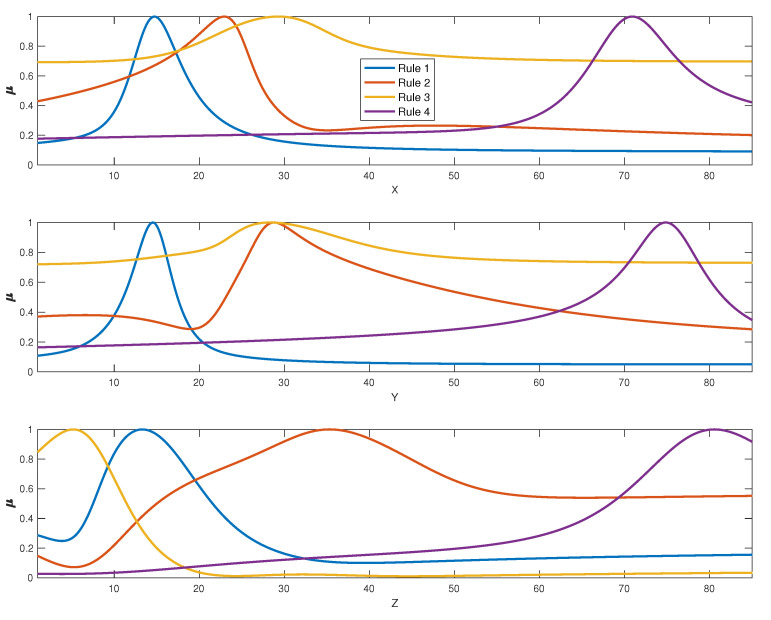
Membership functions for the clusters identified from the CIEXYZ training dataset. Fuzzy membership degree of each data to each fuzzy set is denoted by μ for simplicity (y axis).

This gives us a first glance about the data clustering done but it is also important to have a look at the fuzzy membership functions which are going to be used to determine the degree in which each sample belongs to each of the clusters, which, in turn, will serve as an activation factor for the fuzzy rule used to recover the spectral curve for that cluster and that also provides physical insight behind the method. That is, the memberships we plot in Figure 7 are used to determine the fuzzy degree of membership of each input to the different clusters. These degrees will be used as weights to combine the spectral curves associated to the cluster centers. The spectral curves associated to the cluster centers are plotted in Figure 6. These spectral curves are critical to understanding the physical insight behind the method as they can be seen as the primaries used to recover other spectral curves. By analyzing the cluster membership functions in Figure 7 we can see that membership to cluster 1 is restricted to XYZ data with low values (10–20) in all variables. An XYZ sample can belong to a high degree to cluster 2 if X is very low to low-medium (0–30), Y is medium to high (30–60) and Z is not very low (higher than 20). Cluster 3 is restricted to very low values of Z (0–10) with little importance of the XY values whereas cluster 4 includes only data with high XYZ values. Taking membership functions into account we are able to better understand how the system works: those samples with a non-negligible degree of membership to more than one cluster will be associated to a non-linear combination of spectral curves of the cluster centroids (Figure 6) according to these membership functions.

**Figure 8:**
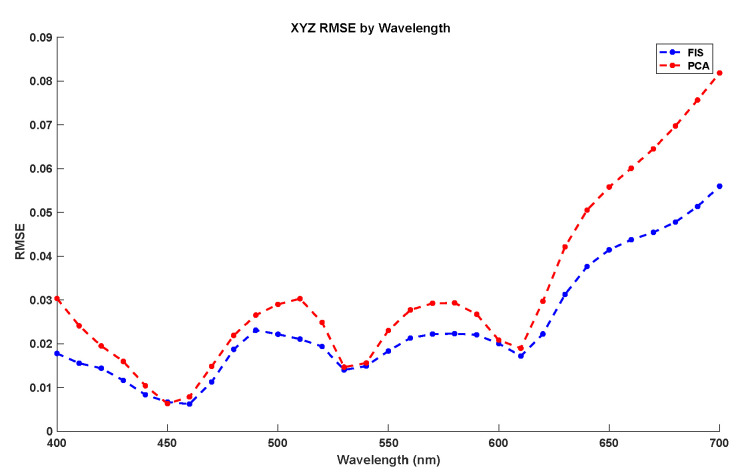
RMSE local error by wavelength comparative between Principal Components Analysis (PCA) and FIS using the CIEXYZ testing dataset.

Last, we compare the global perfomance of the FIS built with the PCA reference method in Table 2 in terms of both mean RMSE error [36] and CIEDE2000 [39] color difference, which should be minimized. RMSE measures the numerical error between the reflectance factors in the recovered and the original curve whereas CIEDE2000 color differences characterize the perceptual error between the colors associated to the recovered and original spectral curves. Notice that for each predicted spectral curve we compute the global error with respect to the desired output to measure performance. Also, we have measured local error (see Figure 8) to particular wavelengths for the best two strategies: PCA and FIS. However, this local performance is not as important as the global performance, as the integration of the spectral reflectance is what is practical in the field of color science: the spectral reflectance recovered is going to be mostly used in different color matching algorithms and in all those algorithms the integration of the spectral reflectance across the visible spectrum is used instead of only some of the wavelengths. Therefore, the local performance of the method is of secondary importance to the global performance of the method. The spectral recovery methods are applied usually in the field of color science where the integration of the spectral curves is important. Human vision is based on a similar procedure as it integrates (summing up) the spectra across the visible spectrum, so the field of color science is mostly focused on the integration of the spectra.

**Figure 9:**
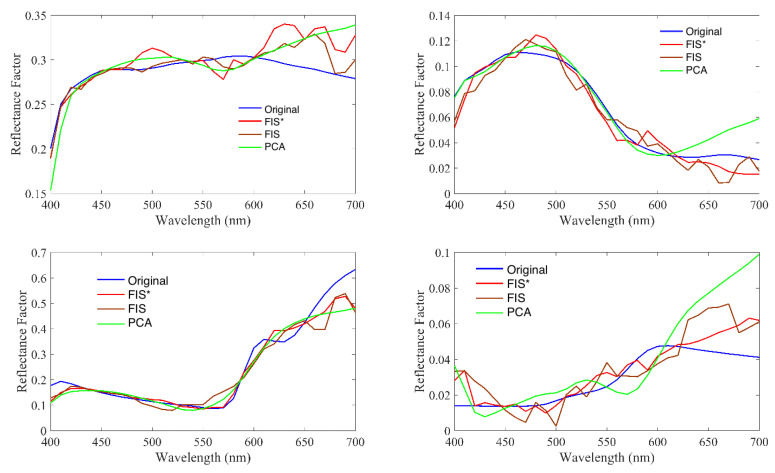
Original and recovered randomly selected testing samples using the recovery methods from CIEXYZ data in the comparison.

From the results in Table 2 and Figure 9, we can see that the global performance of the proposed method outperforms PCA. This is logical as PCA uses linear combinations of three spectral curves to predict the output whereas our method uses four reference spectral curves that are combined in a non-linear way. This implies more flexibility of the whole method, which justifies the improvements obtained. Also, we include in the Table 2 the FIS* variant where the input multiple-clusterization option is used to confirm that improvement is marginal while understanding the system procedure would be too complex in this case.

Figure 8 compares the local prediction error of the different reflectances between the two methods with the best global performance: PCA and FIS. In this figure, we have calculated the RMSE error for each wavelength reflectance estimation for the whole testing dataset. As results show, both methods have a better local performance for low and medium wavelengths, with a quasi-linear increase in the prediction error at high frequencies. Also, for all wavelengths the FIS system has better local performance than PCA.

Also, we show some spectral recovery samples in Figure 9 to illustrate the performance differences between the methods in the comparison. From these plots we can see that despite the recovered error between the spectral curves is low, the recovered curves differ from the original in their smoothness: they are not as smooth as the original. Although from a strictly physical point of view it would make more sense that the recovered curves are more smooth, spectral recovery methods are usually applied in the field of color science to be used in the subsequent color matching algorithms. Using these recovery methods, a metamer curve of the original spectral reflectance is estimated. The curves recovered in Figure 9, are, at least, very close to a metamer to the original curve, which could be concluded by looking at the number of intersects the original and the recovered spectra have. Because the spectral curves of the recovered spectra are going to be used in a color matching or color related application, it is not really critical that the recovered curves are not smooth, as reflectance spectral is going to be integrated across the spectrum anyway. So, there is no need to be worried about the fact that the spectra are not smooth. However, if the spectra were going to be used for material identification or other areas that are not strictly in the field of color science, a post smoothing filter could be applied to the data, as suggested by [40].

### 3.2. Spectral Recovery from Rgb Values

**Figure 10:**
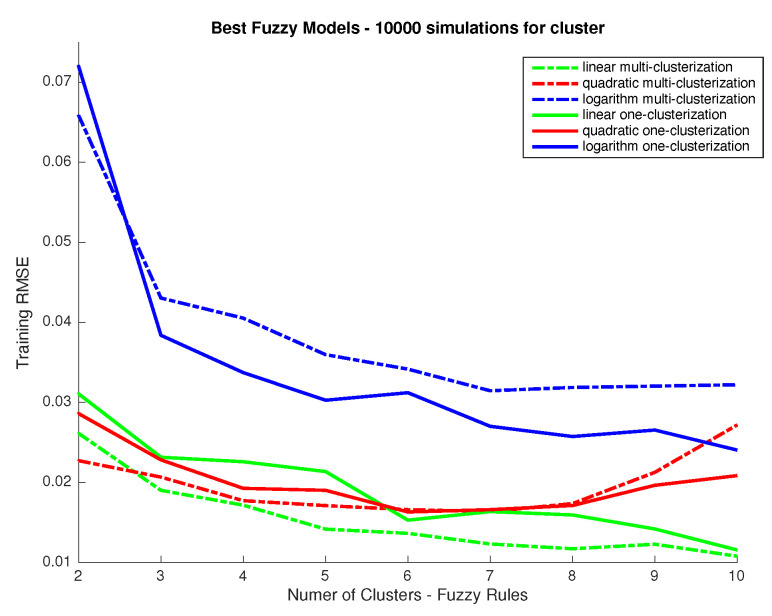
RMSE between predictions from the FIS system and desired outputs in the RGB training dataset (y axis) vs. number of clusters used (x axis). Legend indicates for each plot if the same input clusterization is used for all outputs or not.

**Figure 11:**
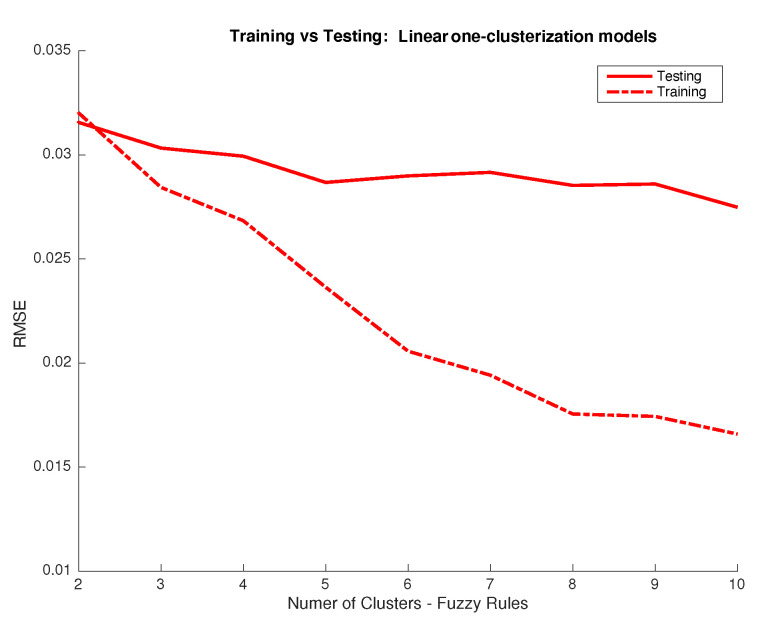
RMSE between predictions from FIS system using the same clusterization for all inputs and linear activation functions and desired outputs in the RGB training and testing datasets (y axis) vs. number of clusters used (x axis).

Now we approach the spectral recovery from RGB data through a trained FIS. Analogously as in the previous section, we first fix the main system parameters: response functions and number of clusters/rules per output. As above, we find that linear response functions provide the best performance for 10,000 training simulations (See Figure 10). Also, improvements for variable number of clusters per output are not significant and we use again the same number of clusters and rules for all outputs. Figure 11 shows the RSME error for the training and validation sets for a different number of clusters per output. We can see that consistent improvements over 1000 simulations are obtained for the validation dataset when increasing the clusters up to 4 or 5. To agree with the previous section, we pick again four clusters and, thus, rules, per output.

Once these parameters are set we train and build the FIS for spectral recovery from RGB data using four clusters per output and linear responses and we analyze the FIS built to explain how it is working.

**Figure 12:**
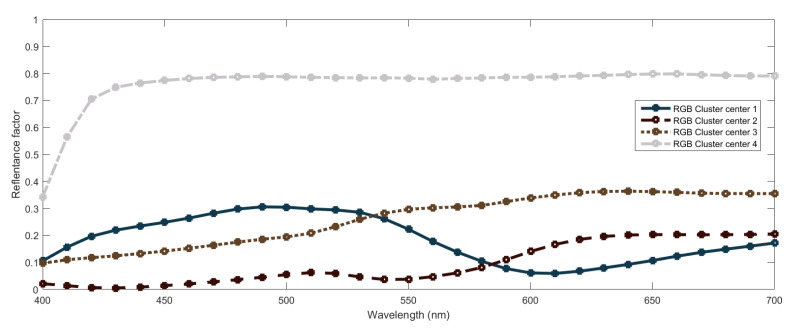
Spectral curves associated to cluster centers obtained in the FIS using RGB data. For each center, the curve is plotted using the RGB coordinates associated to the color represented by the curve itself for illustrative purposes.

Table 3 shows the centers of the four clusters. We can see that center of the first cluster corresponds to a dark cyan, the one of the second to a dark purplish gray, the third to medium orange and the fourth to a light gray. As commented above, the outputs associated to these centers will be used to predict the responses for other very close inputs. We plot these outputs in Figure 12.

**Figure 13:**
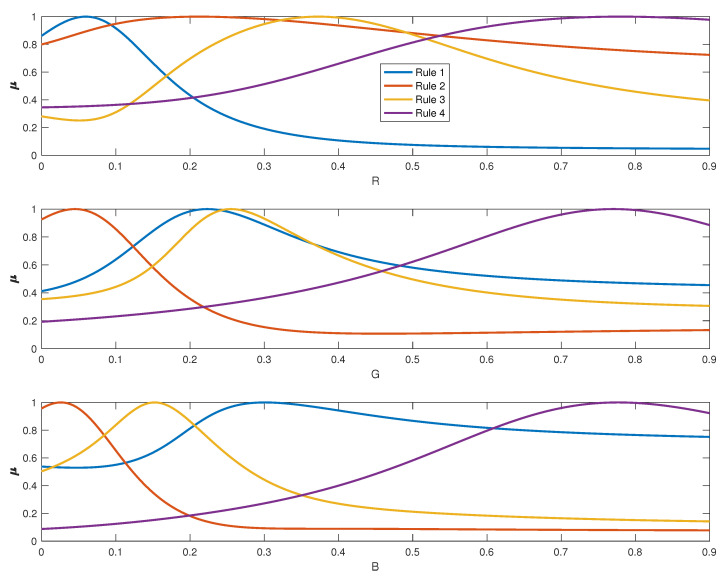
Membership functions for the clusters identified from the RGB training dataset. Fuzzy membership degree of each data to each fuzzy set is denoted by μ for simplicity (y axis).

Next, we look at the membership functions used in the different clusters/rules which we plot in Figure 13 to understand the physical meaning of each cluster and how the spectral curves of the cluster centers are used to obtain other curves. We can see that first cluster mainly concerns colors with a low red component. The second cluster is associated with red colors of different saturation as it includes different ranges of red components with very low G and B components. The third cluster contains colors with a low B component and different amount of R and G, whereas the fourth one concern colors with medium to high RGB components.

Last, we compare the global performance of the FIS built with the Pseudo-Inverse and Cao’s reference methods in Table 4 in terms of both mean RMSE error and CIEDE2000 color difference. We can see that the proposed method significantly outperforms the others. Again, the use of four well-chosen reference spectral curves that are combined in a non-linear way allows to obtain this high performance. Also, we include in the table the FIS* variant where the input multiple-clusterization option is used to confirm that improvement is marginal while understanding the system procedure would be too complex in this case.

**Figure 14:**
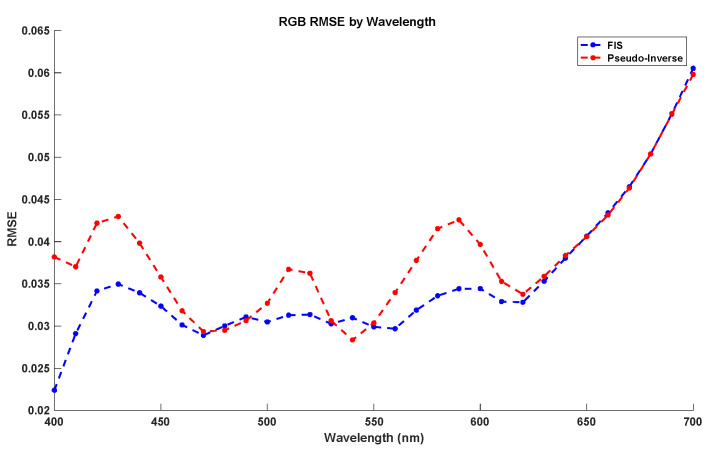
RMSE local error by wavelength comparative between Pseudo-Inverse and FIS using the testing dataset.

In addition, as in the previous section, Figure 14 compares the local prediction error of the different reflectances between the two methods with the best global performance: Pseudo-Inverse and FIS. In this figure, we have calculated the RMSE error for each wavelength reflectance estimation for the whole testing dataset. As results show, both methods have a better local performance for low and medium wavelengths, with a quasi-exponential increase in the prediction error at high frequencies. Furthermore, for almost all wavelengths the FIS system has better local performance than Pseudo-Inverse.

**Figure 15:**
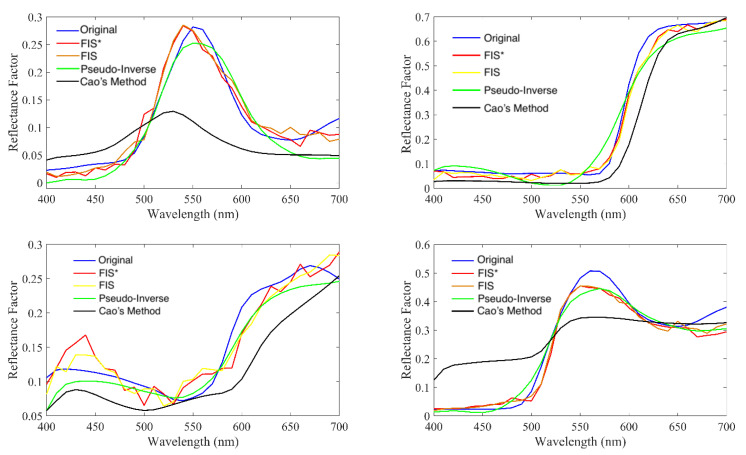
Four randomly selected samples along with their recovered ones using the RGB methods in the comparison.

Finally, we show some spectral recovery samples in Figure 15 to illustrate the performance differences between the methods in the comparison. Comments above about recovered curves smoothness can be made here, as well.

### 3.3. Discussion

It is interesting to point out the similarities and differences of the systems built. Both systems are aimed at recovering spectral curves in an ill-posed problem using similar datasets for training. It is interesting to stress that despite some similarities exist, the systems are not equivalent.

**Figure 16:**
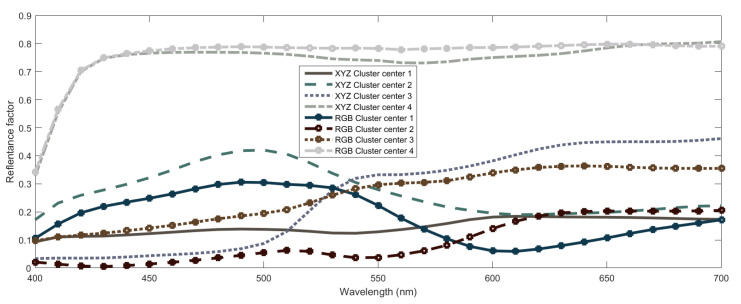
Spectral curves associated to cluster centers obtained in the FIS using both CIEXYZ and RGB data. For each center, the corresponding RGB coordinates are used to plot the curve.

The strongest point of agreement between the two systems concerns that successful spectral recovery can be obtained by using four reference spectral curves that are combined in a non-linear way to predict other spectral curves. Somehow, both systems are using four kinds of *primary* spectral curves to combine and, thus, obtain other curves. For a better comparison, we show in Figure 16 the two sets of four spectral curves used in the systems, where we can see that there is a high similarity between the sets: both systems use a light-gray and a dark-gray like curve, a curve with a higher response in lower wavelengths and another with a higher response in higher wavelengths.

However, the reference spectral curves used by each system are not exactly the same. This is related to the fact that dataset clusterization for CIEXYZ and RGB data are different. This is logical as transformation between these spaces do not fully preserve Euclidean distances between the points which are in turn used to build the clusters. Nevertheless, we can identify some similarities between the clustering obtained through the fuzzy membership functions in each case. For instance, in both systems there is a cluster associated to light gray colors and another cluster with colors with a low B component. Also, cluster one in the CIEXYZ system has a significant overlapping with cluster two of the RGB system and cluster two of the CIEXYZ with cluster three of the RGB. Finally, all of this agrees with the similarities observed between the cluster center’s spectral curves sets.

**Figure 17:**
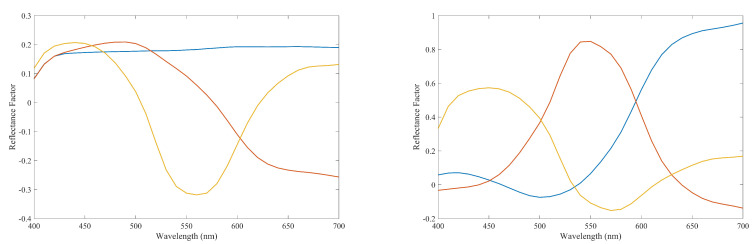
Base functions used by the PCA method (**left**) and Pseudo-Inverse method (**right**).

With respect to the physical meaning of this method and in relation to the methods in the comparison we should point out that PCA and Pseudo-Inverse (PI) use three basis functions to do the spectral reflectance recovery shown in Figure 17. It should be noted that the basis functions used by PCA are the eigenvectors and the basis functions used by PI are simply the matrix of PI containing the relationship between the spectral and RGB information. As it is observed from the Figure 17, the basis functions of both methods contain negative values making them physically impossible to reproduce. In other words, these basis functions are not physically realistic. However, in the case of the FIS, the spectral curves used for the spectral recovery process are all non-negative values, making them physically more realistic. This could be one reason why the results of spectral recovery are better for FIS. Another reason for this could be the fact that four spectral curves are used for the recovery process while in PCA and PI only three basis functions are used. These basis functions and the spectral curves used in FIS somehow act as primaries to match the spectral curves of the testing samples. This larger number of primaries justifies a better spectral recovery result. The non-linear combination of the spectral curves used in FIS is another likely reason for a better result while in PCA and PI a linear combination of the basis functions is used for spectral recovery.

Finally, although it is not aimed to compare the recovery from CIEXYZ to that from RGB, it is worth noting that the former has led to a better recovery accuracy. This totally makes sense, considering the fact that CIEXYZ is a standard color space and is closer to the spectral information than the RGB space, which is a nonstandard color space where other uncertainties, such as random noise, can be found.

## 4. Conclusions

In this work, we approach the problem of spectral reflectance curve recovery from CIEXYZ and RGB data. Instead of building a Fuzzy Logic Inference System (FIS) from expert knowledge we have used a software for training a FIS from CIEXYZ and spectral data and another from RGB and spectral ground truth data, which constitutes the first application of fuzzy logic in such problems. We have conducted extensive simulations to decide the parameter setting of the system. Once the systems were built, we analyzed the obtained results in order to give a physical meaning to the recovery operation made by each of them. We found that both systems use four reference spectral curves that are combined in a non-linear way to predict other spectral curves. Somehow, two kinds of *primary* spectral curve sets are combined to obtain others and the sets found share some similarities. We compare the performance in terms of RMSE and CIEDE2000 difference to show that the systems obtained outperform other methods in the state of the art. Finally, it is interesting to point out that our approach of FIS training and knowledge extraction serves also as an example of a strategy that could be interesting to use in other problems in color and spectral science where it may be interesting to extract knowledge from ground truth datasets. 

## Figures and Tables

**Table 1:** Cluster centers for CIEXYZ values.

Rule/Cluster	X	Y	Z
1	1.4×10^e+1^	1.4×10^e+1^	1.3×10^e+1^
2	2.2×10^e+1^	2.8×10^e+1^	3.5×10^e+1^
3	2.9×10^e+1^	2.8×10^e+1^	5.1×10^e+0^
4	7.0×10^e+1^	7.4×10^e+1^	8.0×10^e+1^

**Table 2:** Performance in spectral recovery from CIEXYZ for the reference PCA method and the build FIS method. We also include the FIS* variant where outputs multi-clusterization option is considered.

Method	Mean *RMSE*	Mean CIEDE2000
PCA	0.0311	2.46
FIS	0.0221	1.85
FIS*	0.0220	1.83

**Table 3:** Cluster centers for RGB values.

Rule	R	G	B
1	5.9×10^e−2^	2.2×10^e−1^	3.0×10^e−1^
2	2.1×10^e−1^	4.4×10^e−2^	2.5×10^e−2^
3	3.7×10^e−1^	2.5×10^e−1^	1.5×10^e−1^
4	7.8×10^e−1^	7.7×10^e−1^	7.7×10^e−1^

**Table 4:** Performance in spectral recovery from RGB for the reference methods and the build FIS method. We also include the FIS* variant where the outputs multi-clusterization option is considered.

Method	Mean *RMSE*	Mean CIEDE2000
Pseudo-Inverse	0.0319	3.96
FIS	0.0281	3.30
FIS*	0.0278	3.35
Cao’s method	0.0791	9.30
